# Long-Read HiFi Genome Sequencing Resolves Retrotransposon-Mediated Deletions in TANGO2 Deficiency Disorder

**DOI:** 10.1212/NXG.0000000000200377

**Published:** 2026-04-29

**Authors:** Quentin Sabbagh, Felipe Villa Tobón, Zahra Kazemi, Laura Lentini, Marie-Emmanuelle Dilenge, Daniela Buhas, Tomi Pastinen, Isabelle Thiffault, Geneviève Bernard

**Affiliations:** 1 Department of Medical Genetics, Reference Center for Developmental Defects and Malformation Syndrome (AnDDI-Rares Network), Montpellier University Hospital, Montpellier University, France;; 2 French Society for Predictive and Personalized Medicine (SFMPP), Montpellier, France;; 3 Child Health and Human Development Program, Research Institute of the McGill University Health Centre, Montréal, Quebec, Canada;; 4 Department of Neurology and Neurosurgery, McGill University, Montréal, Quebec, Canada;; 5 Department of Human Genetics, McGill University, Montréal, Quebec, Canada;; 6 Department of Specialized Medicine, Division of Medical Genetics, Montreal Children's Hospital and McGill University Health Centre, Quebec, Canada;; 7 Genomic Medicine Center, Department of Pediatrics, Children's Mercy Kansas City, MO;; 8 UKMC School of Medicine, University of Missouri Kansas City;; 9 Department of Pathology and Laboratory Medicine, Children's Mercy Kansas City, MO; and; 10 Department of Pediatrics, McGill University, Montréal, Quebec, Canada.

## Abstract

**Background:**

TANGO2 deficiency disorder (TDD) is a rare autosomal recessive condition characterized by neurodevelopmental delay, epilepsy, and metabolic crises, mainly caused by recurrent deletions in *TANGO2*. This study reports the identification of TDD in 2 unrelated families using long-read HiFi genome sequencing (GS), which uncovered homozygous deletions missed by conventional next-generation sequencing (NGS).

**Methods:**

Long-read HiFi GS was performed on blood-derived genomic DNA from affected individuals in both families using the Revio system (Pacific Biosciences).

**Results:**

Homozygous deletions spanning exons 3–9 in family 1 and exons 4–6 in family 2 were identified. These recurrent deletions resulted from genomic recombination between an *Alu* element and distinct retrotransposon subclasses: ERV1 in family 1 and L1MB8 in family 2. The lack of extended homology at breakpoint junctions, deletion sizes, and the dense repetitive genomic architecture support a replication-based rearrangement mechanism, most consistent with fork stalling and template switching or microhomology-mediated break-induced repair (FoSTeS/MMBIR).

**Discussion:**

This study highlights the diagnostic value of long-read HiFi GS for detecting structural variants (SVs) overlooked by standard-of-care NGS and enabling base-pair level breakpoint characterization. It provides the first evidence of retrotransposon-mediated recombination causing recurrent *TANGO2* deletions, elucidating the mechanistic underpinnings of genomic instability at this locus. Finally, these results suggest that TDD prevalence may be underestimated because of undetected SVs.

## Introduction

TANGO2 deficiency disorder (TDD, Mendelian inheritance in man [MIM] 616878) is a rare autosomal recessive condition with early-childhood onset, caused by biallelic pathogenic variants in *TANGO2*.^1,2^ While the exact intracellular function of TANGO2 remains unclear, studies suggest that its deficiency disrupts endoplasmic reticulum-to-Golgi protein trafficking and may lead to secondary mitochondrial dysfunction.^3,4^ Clinically, TDD presents with global neurodevelopmental delay, seizures, dystonia, and hypothyroidism. Affected individuals may also undergo life-threatening episodic metabolic crises marked by acute neurologic deterioration, rhabdomyolysis, and ventricular arrhythmias, often triggered by environmental stressors such as febrile infections or prolonged fasting.^2,5-7^ Genotypically, recurrent *TANGO2* deletions account for most TDD cases (58.2%), with the most common deletions involving exons 3–9 (49.3%) and exons 4–6 (5.5%), as shown in a recent cohort study of 73 TDD individuals.^7^ In this study, we report the diagnostic resolution of TDD in 2 unrelated families using long-read HiFi genome sequencing (GS), which identified homozygous deletions of *TANGO2* exons 3–9 (family 1) and 4–6 (family 2) initially missed by conventional genetic testing. Moreover, the long-read sequencing approach allowed for a detailed analysis of the breakpoint regions, uncovering retrotransposon-mediated genomic recombination as the likely mechanism driving these recurrent deletions.

## Methods

### Long-Read HiFi GS

Long-read HiFi GS was performed on blood-derived genomic DNA using the SMRTbell Express Template Prep Kit 3.0 (Pacific Biosciences), as described in previous studies.^8^ Samples were sequenced on the Revio system, aligned to GRCh38 assembly, and analyzed with Via software (Bionano Genomics) for SNVs and structural variants. Detailed protocols for both the long-read HiFi GS and the standard-of-care short-read exome sequencing (ES) are provided in eMethods 1–2.

### Breakpoint Analysis

Chimeric read sequences of both *TANGO2* deletions were extracted from long-read HiFi GS binary alignement map (BAM) files and manually inspected. Repetitive elements at the breakpoints were retrieved using RepeatMasker data set.

### Standard Protocol Approvals, Registrations, and Patient Consents

Clinical examination and NGS-based genetic testing were conducted as part of routine care, while long-read HiFi GS was performed in research settings. Written informed consent for participation in the research and publication was obtained from all individuals or their legal guardians. The study was approved by the Research Ethics Boards of Montreal Children's Hospital (no. 11-105-PED) and McGill University Health Centre (no. 2019-4972).

### Data Availability

Data not provided in the article because of space limitations may be shared (pseudonymized) at the request of any qualified investigator for purposes of replicating procedures and results.

## Results

### Clinical Description and Genetic Testing

#### Family 1

The proband (II-1) is a female born at term with normal parameters to unrelated, healthy parents. Early signs included global developmental delay and severe behavioral issues with self-injury, later improving. She walked and spoke her first words at 25 months, but at 7 years, expressive language remained limited to 20 words. At 20 months, she experienced a 36-hour dystonic episode after a pneumonia. Subsequent paroxysmal events included alternating hemiparesis, dystonia exacerbation, and staring spells. Moreover, she was diagnosed with hypothyroidism at 7 years and focal-onset epilepsy at 9 years. By age 19, she had moderate-to-severe dysarthria and had lost ambulation, using a walker occasionally, because of severe ataxia, generalized dystonia, and spasticity. Severe dysphagia necessitated gastrostomy placement at 17 years, while brain MRIs at 1 and 4 years were unremarkable. A detailed clinical description is provided in Table 1.

**Table 1:** Clinical Features of Affected Individuals From Both Reported Families

	Family 1	Family 2			
Family member	Proband (II-1)	Proband (II-5)	Brother (II-1)	Sister (II-2)	Sister (II-3)
Pathogenic *TANGO2* variant	Exon 3–9 deletion	Exon 4–6 deletion	Exon 4–6 deletion	Exon 4–6 deletion	Exon 4–6 deletion
Geographical origin	North American	South Asian	South Asian	South Asian	South Asian
Age at disease onset	20 mo	15 mo	1 mo	2 d	3 mo
Onset-associated symptom(s)	TANGO2 spell	Seizures	Seizures	Acute metabolic decompensation	Seizures
Status	Alive	Alive	Deceased	Deceased	Deceased
Age at time of death	N/A	N/A	5 y 6 mo	5 mo	21 mo
Developmental delay	Yes	Yes	Yes	Yes	Yes
Independent walking achieved	Yes	No	No	Too young to evaluate	No
Age at achievement	25 mo	N/A (walking with support at 3 y)	N/A	N/A	N/A
Speech/language delay	Yes	Yes	Unknown	No	Unknown
Description	First spoken words at 25 mo	First spoken words at 24 mo	N/A	N/A	N/A
Cognitive impairment	Yes	Yes	Unknown	Too young to evaluate	Too young to evaluate
Developmental regression	Yes	Yes	N/A	Yes	Yes
Age at last evaluation	19 y	14 y	5 y 6 mo	5 mo	1 y 9 mo
Functional scores at last evaluation					
Gross motor function classification system (GMFCS)	Level IV	Level V	Level V	Level V	Level V
Manual ability classification system (MACS)	Level V	Level V	Level V	Too young to evaluate	Level V
Eating and drinking classification system (EDAC)	Level V	Level V	Level V	Too young to evaluate	Level V
Communication function classification system (CFCS)	Level IV	Level V	Level V	Too young to evaluate	Too young to evaluate
Failure to thrive	No	No	Yes	Yes	Yes
Low growth parameters	No	No	Yes	Yes	Yes
Microcephaly	Yes	Yes	Yes	Yes	Yes
Behavioral issues	Yes (self-mutilation)	No	No	No	No
Epilepsy	Yes	Yes	Yes	No	Yes
Dysarthria	Yes	Yes	Yes	Too young to evaluate	Too young to evaluate
Sialorrhea	Yes	Yes	Unknown	Too young to evaluate	Too young to evaluate
Spasticity	Yes	Yes	Yes	Yes	Yes
Axial hypotonia	Yes	Yes	Yes	Yes	Yes
Extrapyramidal signs	Yes	Yes	Unknown	No	No
Description	Generalized dystonia	Generalized dystonia	N/A	N/A	N/A
Cerebellar ataxia	Yes	Yes	Unknown	Too young to evaluate	Too young to evaluate
Hypothyroidism	Yes	Yes	No	Unknown	Unknown
Optic atrophy and/or visual loss	No	Yes	Yes	Yes	Unknown
Strabismus	Yes	No	Yes	Yes	No
Description	Intermittent right exotropia	N/A	Exotropia	Exotropia	N/A
Sensorineural hearing loss	No	No	Yes	Yes	Unknown
Dysphagia	Yes	Yes	Yes	Yes	Yes
Age at gastric tube feeding	16 y (gastrostomy)	6 y 4 mo (gastrostomy)	5 y 4 mo (nasogastric tube)	4 mo (nasogastric tube)	1 y 6 mo (nasogastric tube)
TANGO2 spells	Yes	No	No	No	No
Age at first spell	20 mo	N/A	N/A	N/A	N/A
Triggered by	Respiratory infection	N/A	N/A	N/A	N/A
Hypotonia/loss of muscle control	Yes	N/A	N/A	N/A	N/A
Dystonia	Yes (generalized)	N/A	N/A	N/A	N/A
Dysarthria	Yes	N/A	N/A	N/A	N/A
Altered level of consciousness	Yes	N/A	N/A	N/A	N/A
Duration of spell	36 h	N/A	N/A	N/A	N/A
Acute metabolic decompensations	Yes	Yes	No	Yes	Yes
Triggered by	Unclear	EBV infection	N/A	Unknown	Respiratory infection
Rhabdomyolysis	Unknown	Yes (with acute kidney failure)	N/A	Unknown	Unknown
Hypoglycemia	Yes	No	N/A	Yes	Unknown
Lactic acidosis	Yes	No	N/A	Yes	Yes
Ketoacidosis	Yes	No	N/A	No	Unknown
Cardiac crisis	No	No	N/A	No	No (but pericarditis with H1N1 infection)

Abbreviation: N/A = not applicable.

TANGO2 spells correspond to transient, non–life-threatening paroxysmal neurologic events. In contrast, cardiac crises are characterized by the occurrence of ventricular arrhythmias, cardiomyopathy (manifesting as heart failure), and/or cardiac arrest, typically in the context of an acute metabolic decompensation.

**Figure 1:**
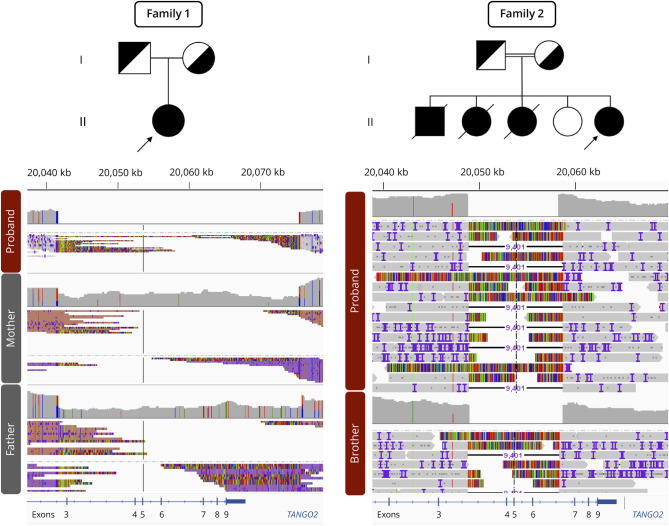
Pedigree of Reported Families and Long-Read HiFi Genome Sequencing Results In family 1, Integrative Genomics View ever (IGV) snapshot shows a homozygous deletion encompassing *TANGO2* exons 3–9, identified in the proband (II-1) by trio long-read HiFi genome sequencing (GS). This deletion removes the stop codon of the gene, supporting a loss-of-function effect. In family 2, IGV snapshot reveals a homozygous in-frame deletion spanning *TANGO2* exons 4–6, detected in the proband (II-5) and her affected, deceased older brother (II-1) through duo long-read HiFi GS. Because exons 3 and 7 are expected to remain in frame, this deletion might produce a smaller, nonfunctional protein or alternatively cause abnormal splicing leading to loss-of-function. For both deletions, characteristic soft-clipped reads are observed along with a complete loss of coverage in homozygous individuals.

Trio ES and chromosomal microarray (CMA) were nondiagnostic. Long-read HiFi GS was then conducted in a trio approach and revealed a homozygous pathogenic deletion spanning exons 3–9 of *TANGO2* (chr22:g.20041611-20075431del [GRCh38]), with parental heterozygosity (Figure 1).

#### Family 2

The proband (II-5), a female born at term to consanguineous parents, had microcephaly, low birth weight (both −2.5 SD), early global developmental delay, and axial hypotonia. She developed refractory epilepsy at 15 months and progressive dysphagia, which necessitated gastrostomy placement at 6 years. At 8 years, she was hospitalized for rhabdomyolysis, transaminitis, and acute kidney failure during an Epstein-Barr virus (EBV)-confirmed respiratory infection. Neurodevelopmental regression led to the loss of motor and communication abilities at 13 years. At 14, she became nonambulatory with axial hypotonia, spastic quadriparesis, generalized dystonia, and cerebellar ataxia. Brain MRIs showed progressive cerebral and cerebellar atrophy along with the most recent showing bilateral pallidal mineral deposition. Three siblings showed overlapping phenotypes with neurodevelopmental delay, regression, seizures, spasticity, axial hypotonia, optic atrophy, and low growth parameters. The eldest brother had a slowly progressive course, while the 2 sisters exhibited a more severe progression with acute decompensations and metabolic crises during infections. All 3 died of respiratory complications at 5 months (II-2), 21 months (II-3), and 5 years (II-1). A detailed clinical description of the proband and her affected siblings is provided in Table 1.

Genetic testing, including trio ES and CMA, was nondiagnostic in the proband. Similarly, her deceased brother underwent noncontributory trio ES. Long-read HiFi GS was then performed in a duo approach in the proband and her deceased brother, identifying a homozygous pathogenic deletion spanning exons 4–6 of *TANGO2* (chr22:g.20048861-20058261del [GRCh38]) (Figure 1).

### Breakpoint Characterization

**Figure 2:**
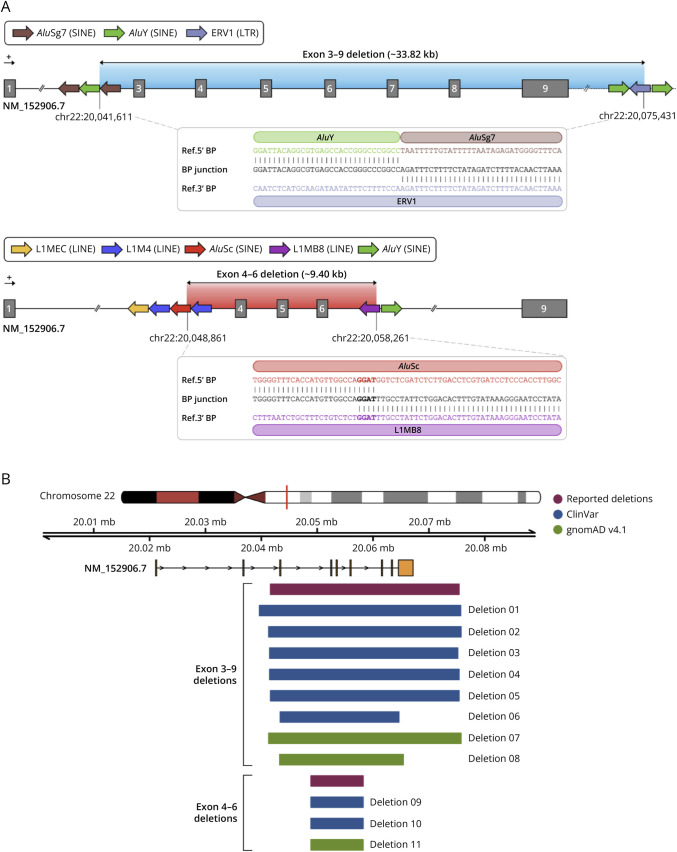
Breakpoint Characterization of *TANGO2 *Deletions Observed in This Study and Comparison With Previously Reported Events (A) The exons 3–9 deletion (∼33.82 kb) in family 1 results from retrotransposon-mediated recombination, with fusion of the upstream *Alu*Y element and downstream ERV1 sequence at the breakpoint (BP) junction (upper panel). The exons 4–6 deletion (∼9.40 kb) in family 2 also involves retrotransposon-mediated recombination, with fusion of the upstream *Alu*Sc element and downstream L1MB8 sequence, along with a four-nucleotide microhomology at the BP junction (highlighted in bold; lower panel). (B) Breakpoint mapping for the *TANGO2* deletions reported in this study and comparison with corresponding events from ClinVar and gnomAD v4.1 data sets. Genomic coordinates and gnomAD population frequencies are provided in eTables 1 and 2. Annotation was conducted using GRCh38 genome assembly, while *TANGO2* MANE Select transcript (NM_152906.7) was used as reference for both schematic illustrations. LINE = long interspersed nuclear element; LTR = long terminal repeat; SINE = short interspersed nuclear element.

The reported *TANGO2* exons 3–9 (∼33.82 kb) and exons 4–6 (∼9.40 kb) deletions both involved retrotransposon-mediated genomic rearrangements. The exons 3–9 deletion displays a 5′ breakpoint at the last base of an *Alu*Y sequence, contiguous with an *Alu*Sg7 element, in intron 2, and a 3′ breakpoint at an ERV1 sequence downstream, outside the gene's open reading frame (Figure 2A). The exons 4–6 deletion features a 5′ breakpoint within an *Alu*Sc sequence in intron 3, adjacent to a L1M4 element, with the 3′ breakpoint in intron 6 at a L1MB8 sequence (Figure 2B). In both cases, long-read HiFi sequencing revealed fusion of these repetitive sequences at the breakpoint junction. The exons 4–6 deletion further shows a four-nucleotide microhomology at the breakpoint, whereas no microhomology was observed for the exons 3–9 deletion.

## Discussion

The 2 pathogenic deletions reported here involve recombination between *Alu* elements and distinct classes of retrotransposons (*Alu*/ERV1 and *Alu*/LINE), lack or show minimal microhomology at breakpoint junctions, and display recurrent occurrence (Figure 2B). These features, together with the moderate deletion sizes (∼33.82 kb and ∼9.40 kb) and the local enrichment of retrotransposons around breakpoints, support a replication-based mechanism.^9,10^ In this context, FoSTeS/microhomology-mediated break-induced repair emerges as the most plausible hypothesis, because it is promoted by replication stress in repeat-rich genomic regions and does not require extended sequence homology between recombining elements.^11,12^

TDD is characterized by recurrent intragenic deletions involving diverse exon combinations (e.g., 1–2, 3–5, 3–9, 4–6, 4–9),^1,2,7^ consistent with a replication-based mechanism and suggesting a rearrangement hotspot. Our data reveal that the 2 most prevalent *TANGO2* deletions result from retrotransposon-mediated recombination, elucidating the mechanistic underpinnings of genomic instability at this locus. Moreover, documented deletions spanning exons 3–9 exhibit variable breakpoints coordinates (Figure 2B, eTable 1) and occur across a broad genetic ancestry spectrum, predominantly in European non-Finnish populations (gnomAD v4.1; eTable 2). In addition, the exons 4–6 deletion has been identified in heterozygous healthy individuals of South Asian and European descent (eTable 2), as well as in a homozygous affected individual of Middle Eastern ancestry.^1^ Collectively, these findings support a model whereby recurrent *TANGO2* deletions arise through independent, de novo rearrangement events, rather from a single founder effect, underscoring the contribution of retrotransposon-mediated recombination to genomic instability at this locus.

While ES remains a cornerstone of rare disease diagnostics, it failed to detect both recurrent *TANGO2* deletions reported here. This limitation stems from the reliance of short-read copy number variant detection algorithms on 3 key indirect signals—read depth across genomic bins, discordant read pairs, and soft-clipped reads—which can be ambiguous or insufficient for detecting single-gene CNVs, particularly when breakpoints lie within poorly covered regions. Although ES can effectively identify CNVs in well-defined genomic intervals, both reported deletions evaded automated detection and were only revealed through retrospective manual inspection of BAM files (eFigure 1).

In light of these findings, the prevalence of TDD may be underestimated because of recurrent deletions overlooked by standard-of-care next-generation sequencing (NGS). Therefore, we recommend that visual inspection of the *TANGO2* locus be systematically considered in individuals with the characteristic combination of neurodevelopmental delay or epilepsy, hypothyroidism, and episodes of acute metabolic decompensation when NGS analyses remain inconclusive.

In conclusion, this study highlights the added value long-read HiFi GS, which not only enabled the detection of these recurrent *TANGO2* deletions—thereby ending the diagnostic odyssey for both families—but also facilitated base-pair level breakpoint characterization. These findings align with broader observations from the European solving the unsolved rare diseases (SOLVE-RD) initiative, where long-read HiFi GS provided a genetic diagnosis in 11.8% of previously unsolved families.^13^
